# Glycated Haemoglobin A1c Variability Score Elicits Kidney Function Decline in Chinese People Living with Type 2 Diabetes

**DOI:** 10.3390/jcm11226692

**Published:** 2022-11-11

**Authors:** Yiling Zhou, Hongmei Huang, Xueqin Yan, Simona Hapca, Samira Bell, Furong Qu, Li Liu, Xiangyang Chen, Shengzhao Zhang, Qingyang Shi, Xiaoxi Zeng, Miye Wang, Nan Li, Heyue Du, Wentong Meng, Baihai Su, Haoming Tian, Sheyu Li, on behalf of the WECODe Study Group

**Affiliations:** 1 Department of Endocrinology and Metabolism, West China Hospital, Sichuan University, Chengdu 610041, China; 2 Department of Endocrinology and Metabolism, The First People’s Hospital of Shuangliu District, Chengdu 610200, China; 3 Department of Chronic Disease Management, Pidu District Second People’s Hospital, Chengdu 610000, China; 4 Division of Computing Science and Mathematics, University of Stirling, Stirling FK9 4LA, UK; 5 Division of Population Health Science and Genomics, School of Medicine, University of Dundee, Dundee DD2 4BF, UK; 6 Department of General Practice, West China Hospital, Sichuan University, Chengdu 610041, China; 7 Department of Endocrinology and Metabolism, Second People’s Hospital of Ya’an City, Ya’an 625000, China; 8 Department of Pharmacy, Karamay Central Hospital, Karamay 834000, China; 9 Department of Pharmacy, West China Hospital, Sichuan University, Chengdu 610041, China; 10 Chinese Evidence-Based Medicine Center, Cochrane China Center, MAGIC China Center, West China Hospital, Sichuan University, Chengdu 610041, China; 11 Department of Nephrology, West China Hospital, Sichuan University, Chengdu 610041, China; 12 West China Biomedical Big Data Center, West China Hospital, Sichuan University, Chengdu 610041, China; 13 Department of Informatics, West China Hospital, Sichuan University, Chengdu 610041, China; 14 Laboratory of Stem Cell Biology, State Key Laboratory of Biotherapy, West China Hospital, Sichuan University, Chengdu 610041, China

**Keywords:** HbA1c variability, eGFR slope, type 2 diabetes, kidney function decline, electronic medical records

## Abstract

Our aim was to investigate the association of glycated haemoglobin A1c (HbA1c) variability score (HVS) with estimated glomerular filtration rate (eGFR) slope in Chinese adults living with type 2 diabetes. This cohort study included adults with type 2 diabetes attending outpatient clinics between 2011 and 2019 from a large electronic medical record-based database of diabetes in China (WECODe). We estimated the individual-level visit-to-visit HbA1c variability using HVS, a proportion of changes in HbA1c of ≥0.5% (5.5 mmol/mol). We estimated the odds of people experiencing a rapid eGFR annual decline using a logistic regression and differences across HVS categories in the mean eGFR slope using a mixed-effect model. The analysis involved 2397 individuals and a median follow-up of 4.7 years. Compared with people with HVS ≤ 20%, those with HVS of 60% to 80% had 11% higher odds of experiencing rapid eGFR annual decline, with an extra eGFR decline of 0.93 mL/min/1.73 m^2^ per year on average; those with HVS > 80% showed 26% higher odds of experiencing a rapid eGFR annual decline, with an extra decline of 1.83 mL/min/1.73 m^2^ per year on average. Chinese adults with type 2 diabetes and HVS > 60% could experience a more rapid eGFR decline.

## 1. Introduction

People living with type 2 diabetes face an extra risk of premature death and disability from chronic kidney disease (CKD) [1], which initiates from one-way kidney function decline and pushes some people to kidney failure and maintenance kidney replacement therapy (KRT) [2]. Identifying individuals with a more rapid decline of kidney function facilitates personalised prevention of CKD in practice. The estimated glomerular filtration rate (eGFR) slope is a recently validated surrogate outcome supporting the quantitative description of the kidney function decline [3,4,5,6,7] and making this personalised identification possible.

The treatment of type 2 diabetes requires periodic measurements of glycated haemoglobin A1c (HbA1c) to help decide the appropriate treatment for glucose control. In addition to the point-to-point average glucose monitoring, the Veterans Affairs guideline suggests the clinical relevance of intraindividual visit-to-visit variability of HbA1c over time [8]. High HbA1c variability may result from poor healthcare quality, or personal comorbidities and drug response, linking to adverse outcomes [9]. Several metrics describe HbA1c variability [9,10], but few are translatable to practice due to clinical relevance and computational challenges. The HbA1c variability score (HVS) is simple to calculate, with the percentage of successive HbA1c measures differing by ≥0.5% (5.5 mmol/mol), which reflects long-term changes in glycaemic control. Its effectiveness in predicting new-onset CKD and all-cause death has been demonstrated in British people with type 2 diabetes [11,12]. However, it is currently unclear whether HVS is associated with kidney disease progression, thereby hampering the clinical evaluation of kidney impairment using HVS in people with diabetes. To bridge this gap, our study investigates the association between HVS and kidney function decline in Chinese adults with diabetes, regardless of CKD status.

## 2. Materials and Methods

### 2.1. Study Design and Study Population

The West China Electronic medical record Collaboration Of Diabetes (WECODe) is a large electronic medical record (EMR)-based multicentre database of diabetes, capturing longitudinal EMR data of patients with diabetes in both inpatient and outpatient settings from hospitals in Sichuan Province, China, since January 2011 (Appendix A) [13]. This retrospective cohort study enrolled adults with type 2 diabetes from the WECODe outpatient setting, including those with information before maintenance KRT, with ≥five outpatient visits for at least one year (from the first visit with HbA1c measure to the last) between Jan 2011 and Jun 2019. People were excluded if they had fewer than three serum creatinine measurements or baseline eGFR < 15 mL/min/1.73 m^2^ (calculated using the chronic kidney disease epidemiology collaboration formula [14]).

The individual follow-up started at the index date—the first visit with HbA1c measures—and ended at the last visit with HbA1c or serum creatinine measures. The baseline parameters were captured from 30 days before the index date to one year after. The study calculated the average number of outpatient visits per year to estimate patient adherence [15,16,17].

This study was approved by the ethics committee of West China Hospital, Sichuan University (No. 2021-386; No. 2021-282; No. 2020-968; No. 2020-597). Patient consent was waived for this retrospective study of data from electronic medical records.

### 2.2. Data Collection and Calculation

We adopted the previously described formula to calculate HVS [9,11] and categorised HVS at an interval of 20%, identifying five HVS categories, 0% to 20% as the reference, 20% to 40%, 40% to 60%, 60% to 80%, and above 80%. A pilot description of the eGFR trajectory in our study population (Figure S1) supports using a single linear slope throughout the follow-up duration to calculate the eGFR slope.

We retrieved and linked all prespecified medical data produced in outpatient during the study period from the WECODe, including age, sex, diagnosis summary with free text and ICD-10 codes, and date of visit; the dates and records of medication prescription, including insulin, angiotensin II, receptor blockers/angiotensin-converting enzyme inhibitor (ARB/ACEI), statins, and calcium channel blocker (CCB); and the dates and values of laboratory tests, including HbA1c (ion-exchange high-performance liquid chromatography assays), blood glucose, serum creatinine, and lipid profiles. Hypertension or atherosclerotic cardiovascular disease (ASCVD) was identified from the diagnosis summary (Table S1).

### 2.3. Outcomes

The first primary outcome was whether the participant experienced a rapid eGFR annual decline (Yes vs. No), identified by their mean eGFR decline at a rate of 5 mL/min/1.73 m^2^/year or faster during a given time (his/her mean eGFR slope ≤ −5 mL/min/1.73 m^2^/year), which is linked to the high hazard of kidney failure [18]. 

In addition, we estimated the differences across HVS categories in the mean eGFR slope throughout a given time, since previous studies proved a reduction in eGFR slope at 0.75 mL/min/1.73 m^2^ per year predicted an elevated risk of kidney failure [3]. 

### 2.4. Statistical Methods

The baseline characteristics of the overall study population and across HVS categories were described as mean ± standard deviation or median (25% quantile, 75% quantile) for continuous variables, and frequency (percentage) for categorical variables.

The study applied inverse probability weighting with entropy balancing to achieve covariates balance across HVS categories [19]. The entropy balance weights were estimated by weights optimisation under the constraint of the exact balance of covariates’ moments, accounting for age, sex, the baseline eGFR (<60 vs. ≥60 mL/min/1.73 m^2^); whether having comorbidity of hypertension or ASCVD at baseline (Yes vs. No); ever use of insulin, statins, or ARB/ACEI during the follow-up (Yes vs. No); time-weighted average HbA1c throughout the follow-up; and adherence to diabetes management. Figure S2 showed the assessment of the success of covariates balance.

For the first primary outcome, we computed the mean eGFR slope for each individual using least square regression of all measures of eGFR on time throughout his/her whole follow-up. It represents their eGFR declines in a single annual rate from their index date to the end of follow-up on average. We identified participants who experienced a rapid eGFR annual decline in their mean eGFR slope ≤−5 mL/min/1.73 m^2^/year and performed logistic regression with entropy balance weights to obtain the odds ratio (OR) of HVS categories (reference, HVS between 0% to 20%) for it. 

To reduce the variance derived from unreliable estimates, the differences across HVS categories and their 95% confidence intervals (CIs) in the mean eGFR slope throughout the whole follow-up were derived from a linear mixed-effects model with entropy balance weights, including a two-way interaction fixed effect for HVS categories and continuous-time of eGFR (year), and two random effects for intercept and continuous time to account for intra-cluster correlations. 

We performed four subgroup analyses based on sex, age (<60 vs. ≥60 years), ever use of insulin during the follow-up (Yes vs. No), and baseline eGFR (<60 vs. ≥60 mL/min/1.73 m^2^).

To account for the effect of the length of follow-up and number of measures on the mean eGFR slope, we calculated a mean eGFR slope starting from the index date to either 2-year, 3-year, 4-year, and 5-year follow-up. We also performed other sensitivity analyses to assess the robustness of results by excluding individuals with unparallel measures of HbA1c and serum creatinine at the last visit (measure interval ≥ 90 days), by adjusting for baseline HbA1c instead of time-weighted average HbA1c in the calculation of entropy balance weights, by excluding individuals with baseline eGFR < 30 mL/min/1.73 m^2^, and by excluding individuals receiving any prescription of sodium-glucose cotransporter 2 (SGLT2) inhibitors or glucagon-like peptide-1 (GLP-1) receptor agonists during follow up. We adopted 0.75 mL/min/1.73 m^2^ per year as the minimal important difference (MID) for the mean difference of eGFR slope [3]. 

All analyses were conducted using RStudio 2022.7.1.554 (R version 4.2.1). Statistical code for this analysis is freely accessible for any non-commercial reuse at https://github.com/Yiling-Zhou/HVS-and-eGFR-slope (accessed on 8 November 2022).

## 3. Results

This analysis included 2397 patients (Figure S3), with a median follow-up duration of 4.7 years (interquartile, 3.1 to 6.3 years), a median age of 58.5 years, and a median baseline eGFR of 90.4 mL/min/1.73 m^2^. As shown in Table 1, the median outpatient visits were 1.9 times per year, but those in people with HVS > 80% were 1.6 times. The median follow-up time was comparable across HVS categories, ranging from 4.4 to 4.9 years. The median time-weighted average HbA1c was 7.3% (56 mmol/mol) and increased over the HVS getting higher. People with higher HVS showed less comorbidity of hypertension and ASCVD but received more insulin.

**Figure 1:**
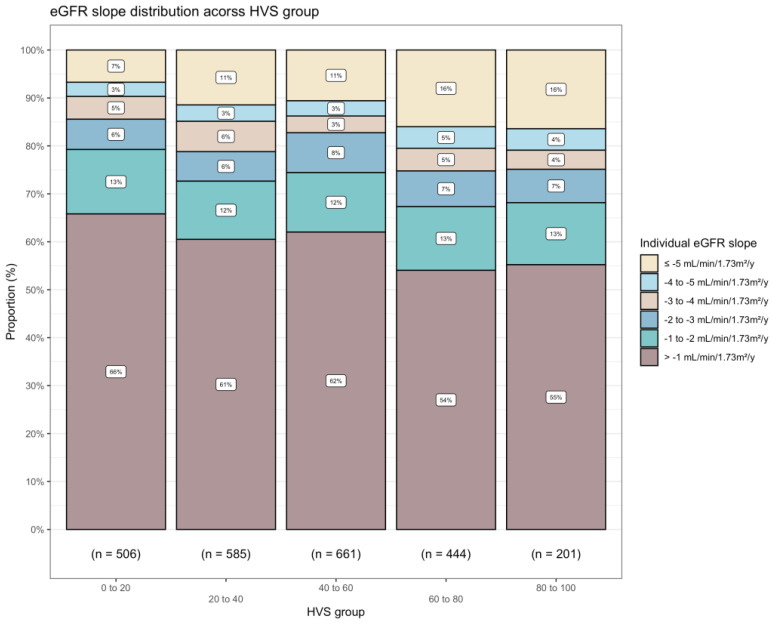
Descriptive analysis for distribution of mean eGFR slope during the whole follow-up in each HVS category. Abbreviations: HVS, glycated haemoglobin A1c variability score; eGFR, estimated glomerular filtration rate. Mean eGFR slope is individual mean eGFR annual change starting from baseline to end of follow-up. The unit of HVS is %.

**Figure 2:**
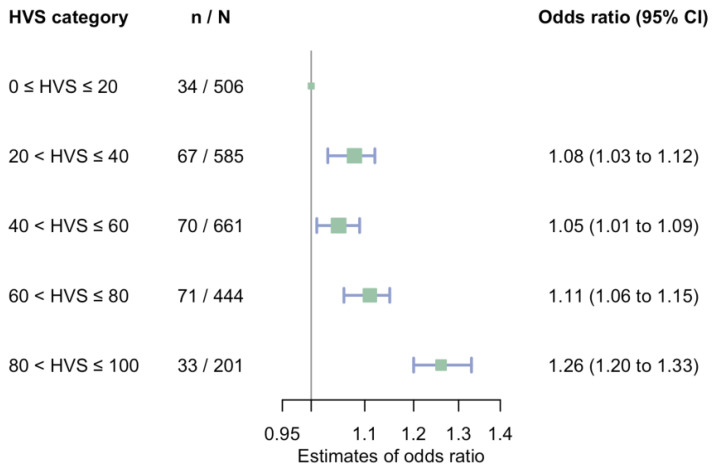
Odds ratio of HVS categories for experiencing a rapid eGFR annual decline. Abbreviations: HVS, glycated haemoglobin A1c variability score; eGFR, estimated glomerular filtration rate; CI, confidence interval. A rapid eGFR annual decline is defined as eGFR annual decline at 5 mL/min/1.73 m^2^/year or more on average from baseline to end of follow-up.

Compared with people with HVS ≤ 20%, those with HVS above 20% face increased odds of annual eGFR slope ≤ −5 mL/min/1.73 m^2^/year (Figure 1 and Figure 2). In people with HVS above 80%, the adjusted OR were 1.26 (95% CI, 1.20 to 1.33, reference, HVS between 0% to 20%) (Figure 2). Figure S4 depicted each subject’s baseline eGFR and mean annual eGFR change from baseline to end of follow-up, stratified by HVS categories. Subgroup analyses identified a potential subgroup effect that people with HVS between 20% and 40% and between 60% and 80% are at a higher risk of a rapid eGFR decline only among those who ever used insulin (Figure S5). Nevertheless, the subgroup effects are not consistent in other HVS categories. 

**Figure 3:**
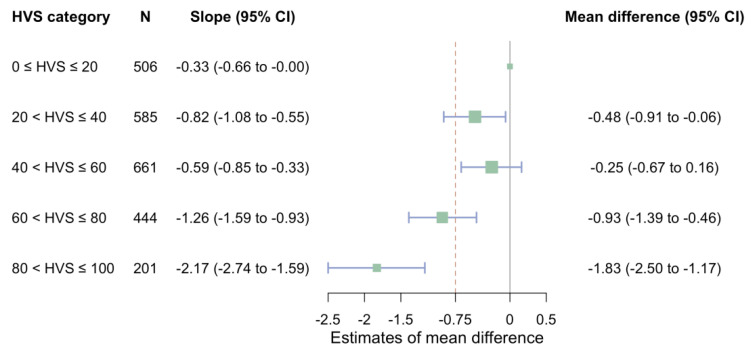
Difference across HVS categories in the mean eGFR slope starting from baseline to end of follow-up. Abbreviations: HVS, glycated haemoglobin A1c variability score; eGFR, estimated glomerular filtration rate; CI, confidence interval.

Compared with people with HVS ≤ 20% whose eGFR declined by 0.33 mL/min/1.73 m^2^ per year on average, the eGFR declined by an extra 0.93 mL/min/1.73 m^2^ (95% CI, 0.46 to 1.39; >MID) per year on average in people with the HVS between 60% and 80% and an extra 1.83 mL/min/1.73 m^2^ (95% CI, 1.17 to 2.50; >MID) per year on average in those with an HVS above 80% (Figure 3). Subgroup analyses indicated that ever use or never use of insulin could modify the difference between people with HVS of 60% to 80% and those with HVS ≤ 20% in the mean eGFR slope but not between those in other HVS categories and HVS ≤ 20% (Figure S6). All sensitivity analyses showed the robustness of the findings (Figures S7–S11). 

## 4. Discussion

To our knowledge, our study firstly showed the association between HVS and kidney function decline in Chinese adults with type 2 diabetes. Those with HVS above 60% face clinically meaningful eGFR decline per year independent of the time-weighted average HbA1c and adherence to diabetes management. Clinicians should take additional attention to the kidney risk of individuals with type 2 diabetes and fluctuating HbA1c values from one visit to another. 

The current paradigm of kidney care in persons with diabetes and CKD is to avoid or delay CKD progression, cardiovascular disease, and the need for dialysis. This framing process begins with the early identification of high-risk individuals, followed by interventions in clinical practice [20]. Previous research has linked HbA1c variability to the risk of kidney failure [21,22,23,24] or eGFR decline [25,26], but their metrics for HbA1c variability (mainly standard deviation or coefficient of variation) are difficult to calculate or interpret by clinicians in their daily practice. HVS, as a recently developed measure for HbA1c variability, is simple to calculate or estimate and easy to interpret clinically, which is instrumental for widespread clinical application [12]. Additionally, the category of the HVS is based on absolute values rather than population quantiles. Using a newly validated parameter and a large database in China, our study bridges the gap between HVS and eGFR slope in Chinese adults with type 2 diabetes. 

The finding of the current study highlights the importance of avoiding fluctuation in blood glucose. Clinicians can calculate the HVS by reviewing HbA1c levels and thereby establishing the proportion of HbA1c change ≥0.5% (5.5 mmol/mol) from the previous read. Individuals with higher HVS are likely to experience rapid kidney function decline and so require additional care. In our study, regardless of their HbA1c on average, adults with type 2 diabetes and almost all HbA1c changes higher than 0.5% or 5.5 mmol/mol (HVS ≥ 80%, 8.3% of the included population) face 26% increased odds of eGFR annual decline higher than 5 mL/min/1.73 m^2^/year, which means an over 12-fold hazard of subsequent kidney failure [18]. In line with previous reports, people with higher HVS have poorly controlled glucose levels and attend appointments less frequently, thereby reflecting the low quality of health care or poor adherence to the care [27,28]. Clinicians should consider more frequent monitoring of kidney function or updating treatment regimens including adding drugs with kidney protection such as SGLT2 inhibitors and RAAS inhibitors [29] for such patients. Patient education is also necessary to improve the adherence to healthcare that may also improve the outcome. 

The physiological mechanism underlying HbA1c variability and kidney function decline remains unclear. Vascular cells exposed to fluctuated glucose produce excess oxidative stress and inflammatory cytokines, which impair the microstructure of the kidneys [30,31,32]. Frequent turnover of hyperglycaemia and hypoglycaemia may modify the epigenetic profiles [33].

This study has several strengths. First, our study utilised a multi-centre dataset, together with multiple sensitivity analyses, yielding a robust result and allowing for its generalisability among people living with type 2 diabetes in Southwest China. Second, this is the first study to explore the association of HVS and eGFR slope in adults living with type 2 diabetes, revealing that an HVS > 60% was associated with a faster kidney function decline, and a higher risk of development of kidney failure. Third, our study performed two primary analyses, complementing each other, to illustrate the HVS could be instrumental to identify patients at a high risk of kidney impairment in clinical practice.

This study does have limitations. First, this observational study could only affirm the association of HVS and eGFR slope, without concluding any causation. Our data call for the exploration of the mechanical studies explaining the phenomenon. Second, the WECODe did not link to the death registry or nationwide discharging system that allows us to explore the association between the HVS and all-cause death and kidney failure. Nevertheless, the newly validated eGFR slope facilitates a possible evaluation of the association of HVS with these patient-important outcomes. Third, a low average number of visits may indicate low adherence to diabetes management and possibly a stable situation of disease control. In this study, however, people with high HVS are unlikely to have stable control, and thus, the association between higher HVS and less frequent visits indicates poorer adherence. 

## 5. Conclusions

This multi-centre study suggests that Chinese adults with type 2 diabetes and HVS above 60% are facing rapid kidney function decline. This information can be vital in assisting clinicians to identify patients at high risk of kidney disease progression, allowing closer attention to implementing strategies to reduce this. 

## Figures and Tables

**Table 1:** Baseline characteristics of the study population.

Characteristics	Overall *n* = 2397	HVS Category				
		0 to 20 *n* = 506	20 to 40 *n* = 585	40 to 60 *n* = 661	60 to 80 *n* = 444	80 to 100 *n* = 201
Age, years	58.5 [48.9, 67.1]	60.3 [52.3, 68.9]	61.2 [51.2, 68.9]	57.5 [47.7, 67.0]	54.9 [45.8, 64.4]	53.4 [43.8, 62.4]
Sex, female, *n* (%)	979 (40.8)	237 (46.8)	238 (40.7)	254 (38.4)	176 (39.6)	74 (36.8)
Follow up, years	4.7 [3.1, 6.3]	4.9 [3.0, 6.6]	4.9 [3.4, 6.7]	4.8 [3.3, 6.2]	4.4 [2.9, 5.8]	4.4 [3.1, 6.0]
Average number of outpatient visits per year, *n*/year	1.9 [1.3, 2.7]	1.9 [1.3, 2.7]	2.0 [1.4, 2.9]	1.9 [1.3, 2.7]	1.8 [1.3, 2.7]	1.6 [1.2, 2.3]
HbA1c, %	7.2 [6.7, 8.3]	6.8 [6.6, 7.1]	7.0 [6.6, 7.9]	7.3 [6.7, 8.7]	7.8 [6.9, 9.1]	8.4 [7.2, 10.3]
HbA1c, mmol/mol	55 [50, 67]	51 [49, 54]	53 [49, 63]	56 [50, 72]	62 [52, 76]	68 [55, 89]
Time-weighted average HbA1c, %	7.3 [6.8, 8.0]	6.8 [6.6, 7.1]	7.1 [6.8, 7.5]	7.4 [7.0, 8.1]	7.8 [7.3, 8.7]	8.6 [7.7, 9.5]
Time-weighted average HbA1c, mmol/mol	56 [51, 64]	51 [49, 54]	54 [51, 58]	57 [53, 65]	62 [56, 72]	70 [61, 80]
eGFR, mL/min/1.73 m^2^	90.4 [74.3, 102.1]	87.8 [73.4, 98.5]	87.8 [72.5, 100.1]	91.4 [75.8, 103.4]	93.8 [77.3, 104.9]	95.5 [78.6, 106.2]
eGFR ≥ 60 mL/min/1.73 m^2^, *n* (%)	2 124 (88.6)	445 (87.9)	507 (86.7)	588 (89.0)	398 (89.6)	186 (92.5)
LDL-c, mmol/L	2.65 [2.03, 3.24]	2.72 [2.09, 3.26]	2.66 [1.92, 3.21]	2.59 [2.06, 3.17]	2.61 [2.01, 3.31]	2.69 [2.02, 3.29]
Hypertension, *n* (%)	1 661 (69.3)	347 (68.6)	444 (75.9)	457 (69.1)	292 (65.8)	121 (60.2)
ASCVD, *n* (%)	954 (39.8)	220 (43.5)	271 (46.3)	251 (38.0)	154 (34.7)	58 (28.9)
Use of insulin, *n* (%)	965 (40.3)	104 (20.6)	207 (35.4)	305 (46.1)	238 (53.6)	111 (55.2)
Use of statins, *n* (%)	1 624 (67.8)	327 (64.6)	408 (69.7)	441 (66.7)	308 (69.4)	140 (69.7)
Use of ARB/ACEI, *n* (%)	1 039 (43.3)	213 (42.1)	274 (46.8)	295 (44.6)	171 (38.5)	86 (42.8)
Use of CCB, *n* (%)	816 (34.0)	173 (34.2)	213 (36.4)	236 (35.7)	136 (30.6)	58 (28.9)

Abbreviations: HVS, glycated haemoglobin A1c variability score; HbA1c, glycated haemoglobin A1c; eGFR, estimated glomerular filtration rate; LDL-c, low-density lipoprotein; ASCVD, atherosclerotic cardiovascular disease; ACEI, angiotensin-converting enzyme inhibitor; ARB, angiotensin II, receptor blockers; CCB, calcium channel blocker. The unit of HVS is %.

## Data Availability

The datasets generated from electronic medical records during the current study are not publicly available due to the data policy. The statistical code of this study is available for any non-commercial academic reuse at https://github.com/Yiling-Zhou/HVS-and-eGFR-slope.

## Supplementary Materials

The following supporting information can be downloaded at https://www.mdpi.com/article/10.3390/jcm11226692/s1, Table S1: Identification of comorbidities from diagnosis summary using the International Classification of Diseases, 10th Revision (ICD-10) codes, or free text. Figure S1: The eGFR trajectory across different HVS categories. Abbreviations: HVS, glycated haemoglobin A1c score; eGFR, estimated glomerular filtration rate. Figure S2: The covariate balance across HVS categories before and after applying entropy balancing A, assessing the covariate balance for logistic regression of HVS and experiencing a rapid eGFR annual decline. B, assessing the covariate balance for the linear mixed effects model. Abbreviations: HVS, glycated haemoglobin A1c variability score; HbA1c, glycated haemoglobin A1c; eGFR, estimated glomerular filtration rate; ASCVD, atherosclerotic cardiovascular disease; ACEI, angiotensin-converting enzyme inhibitor; ARB, angiotensin II, receptor blockers. Figure S3: The flowchart of the selection of study population. * We recruited adults with type 2 diabetes who had ≥ five outpatient visits for at least one year (from the first visit with HbA1c measure to the last) using electronic medical records of four hospitals from the West China Electronic medical record Collaboration Of Diabetes (WECODe) outpatient setting from 1 January 2011 to 30 June 2019. Abbreviations: HbA1c, glycated haemoglobin A1c; HVS, glycated haemoglobin A1c score; eGFR, estimated glomerular filtration rate; KRT, kidney replacement therapy. Figure S4: Individual baseline eGFR and his/her mean eGFR annual change, stratified by HVS categories. Abbreviations: HVS, glycated haemoglobin A1c variability score; eGFR, estimated glomerular filtration rate. The unit of HVS is %. The white point represents the baseline eGFR. The length of red line represents the mean annual decline from baseline to end of follow-up. The length of blue one is the mean annual rise from baseline to end of follow-up. Figure S5: Subgroup analyses of the association between HVS and experiencing a rapid eGFR annual decline. Abbreviations: HVS, glycated haemoglobin A1c score; eGFR, estimated glomerular filtration rate; CI, confidence interval. A rapid eGFR annual decline is defined as eGFR annual decline at 5 mL/min/1.73 m^2^/year or more on average from baseline to end of follow-up. The unit of HVS is %. Figure S6: Subgroup analyses of difference across HVS categories in the mean eGFR slope starting from baseline to end of follow-up. Abbreviations: HVS, glycated haemoglobin A1c score; eGFR, estimated glomerular filtration rate; CI, confidence interval. The unit of HVS is %. Figure S7: Sensitivity analysis by calculating a mean eGFR slope starting from baseline to either 2-year, 3-year, 4-year, and 5-year follow-up. A, odds ratios of HVS categories for experiencing a rapid eGFR annual decline at 5 mL/min/1.73 m^2^/year or more on average from baseline to either 2-year, 3-year, 4-year, and 5-year follow-up. B, the difference across HVS categories in the mean eGFR slope from baseline to either 2-year, 3-year, 4-year, and 5-year follow-up. Abbreviations: HVS, glycated haemoglobin A1c variability score; eGFR, estimated glomerular filtration rate; CI, confidence interval. The unit of HVS is %. Figure S8: Sensitivity analysis by excluding individuals with the last HbA1c and serum creatinine measured ≥ 90 days apart. A, odds ratios of HVS categories for experiencing a rapid eGFR annual decline at 5 mL/min/1.73 m^2^/year or more on average from baseline to end of follow-up. B, the difference across HVS categories in the mean eGFR slope from baseline to end of follow-up. Abbreviations: HVS, glycated haemoglobin A1c variability score; CI, confidence interval. The unit of HVS is %. Figure S9: Sensitivity analysis by adjusting for baseline HbA1c instead of time-weighted average HbA1c when calculating entropy balance weights. A, odds ratios of HVS categories for experiencing a rapid eGFR annual decline at 5 mL/min/1.73 m^2^/year or more on average from baseline to end of follow-up. B, the difference across HVS categories in the mean eGFR slope from baseline to end of follow-up. Abbreviations: HVS, glycated haemoglobin A1c variability score; CI, confidence interval. The unit of HVS is %. Figure S10: Sensitivity analysis by excluding individuals with baseline eGFR < 30 mL/min/1.73 m^2^ instead of eGFR < 15 mL/min/1.73 m^2^. A, odds ratios of HVS categories for experiencing a rapid eGFR annual decline at 5 mL/min/1.73 m^2^/year or more on average from baseline to end of follow-up. B, the difference across HVS categories in the mean eGFR slope from baseline to end of follow-up. Abbreviations: HVS, glycated haemoglobin A1c variability score; CI, confidence interval. The unit of HVS is %. Figure S11: Sensitivity analysis by excluding individuals receiving any prescription of sodium-glucose cotransporter-2 (SGLT2) inhibitors or glucagon-like peptide-1 (GLP1) receptor agonists during follow up. A, odds ratios of HVS categories for experiencing a rapid eGFR annual decline at 5 mL/min/1.73 m^2^/year or more on average from baseline to end of follow-up. B, the difference across HVS categories in the mean eGFR slope from baseline to end of follow-up. Abbreviations: HVS, glycated haemoglobin A1c variability score; CI, confidence interval. The unit of HVS is %.

## Appendix A

Brief summary of the WECODe. The electronic medical record (EMR)-based multicentre database of diabetes, namely West China Electronic medical record Collaboration Of DiabEtes (WECODe), has captured longitudinal EMR data of patients with diabetes in both inpatient and outpatient settings from hospitals in Sichuan Province, China, since January 2011. WECODe includes inpatients if they (1) attended the inpatient department with a discharge diagnosis according to International Classification of Diseases 10th Revision (ICD-10), including codes E10 to E14, fasting glucose > 7.0 mmol/L, 2-hour blood glucose after 75 g glucose challenge > 11.1 mmol/L, random glucose > 11.1 mmol/L, or glycated haemoglobin A1c (HbA1c) > 6.5% (48 mmol/mol); (2) were ≥ 18 years old; and (3) were Chinese and recruits outpatients if they attended the outpatient department and had a diagnosis of “diabetes” in the free text or ICD-10 codes, including E10 to E14 in the EMR. It links anonymized data from eight sources: EMR, demographic records, medical and discharge summaries, prescription records, surgery records, laboratory records, vital sign records, glucose monitoring records, and diagnosis records. All data are archived in the big data platform at West China Hospital of Sichuan University. Up to now, the database, as an ongoing work, has already obtained data from five hospitals and covered > 491 350 people with diabetes, 193,683 people in the inpatient setting with a median follow-up of 43 days, and 297,667 people in the outpatient setting with a median follow-up of 3.2 years.
