# Supplementary material for: Glycated Haemoglobin A1c Variability Score Elicits Kidney Function Decline in Chinese People Living with Type 2 Diabetes
Source: J Clin Med. 2022 Nov 11;11(22):6692. doi: 10.3390/jcm11226692 (PMC9692466; doi:10.3390/jcm11226692)
Supplement: Supplementary file 1 [file jcm-11-06692-s001.zip › jcm-1998950-supplementary.pdf]

Supplementary materials

Contents

Supplementary Table S1 Identification of comorbidities from diagnosis summary using the International Classification of Diseases, 10th Revision (ICD-10) codes, or free text .. 2

Supplementary Figure S1 The eGFR trajectory across different HVS categories............ 3

Supplementary Figure S2 The covariate balance across HVS categories before and after applying entropy balancing 4

Supplementary Figure S3 The flowchart of the selection of study population 6

Supplementary Figure S4 Individual baseline eGFR and his/her mean eGFR annual change, stratified by HVS categories 7

Supplementary Figure S5 Subgroup analyses of the association between HVS and experiencing a rapid eGFR annual decline 8

Supplementary Figure S6 Subgroup analyses of difference across HVS categories in the mean eGFR slope starting from baseline to end of follow-up 9

Supplementary Figure S7 Sensitivity analysis by calculating a mean eGFR slope starting from baseline to either 2-year, 3-year, 4-year, and 5-year follow-up................................ 10

Supplementary Figure S8 Sensitivity analysis by excluding individuals with the last HbA1c and serum creatinine measured ≥ 90 days apart 12

Supplementary Figure S9 Sensitivity analysis by adjusting for baseline HbA1c instead of time-weighted average HbA1c when calculating entropy balance weights...................... 14

Supplementary Figure S10 Sensitivity analysis by excluding individuals with baseline eGFR < 30 mL/min/1.73m^2 instead of eGFR < 15 mL/min/1.73m^2 16

Supplementary Figure S11 Sensitivity analysis by excluding individuals receiving any prescription of sodium-glucose cotransporter-2 (SGLT2) inhibitors or glucagon-like peptide-1 (GLP1) receptor agonists during follow up.. 18

Supplementary Table S1 Identification of comorbidities from diagnosis summary using the International Classification of Diseases, 10th Revision (ICD-10) codes, or free text

Comorbidities	ICD-10 codes	Free text
**Hypertension**	I10 to I15	Free text relating to hypertension; but excluding free text relating to without hypertension, or with or without a family history of hypertension
**Atherosclerotic cardiovascular disease (ASCVD)**	I20 to I25, I61, I63, I64	This study only evaluates the most frequently occurring diseases of ASCVD, free text describing ischemic heart disease, cerebrovascular disease; but excluding free text showing without ASCVD

Supplementary Figure S1 The eGFR trajectory across different HVS categories

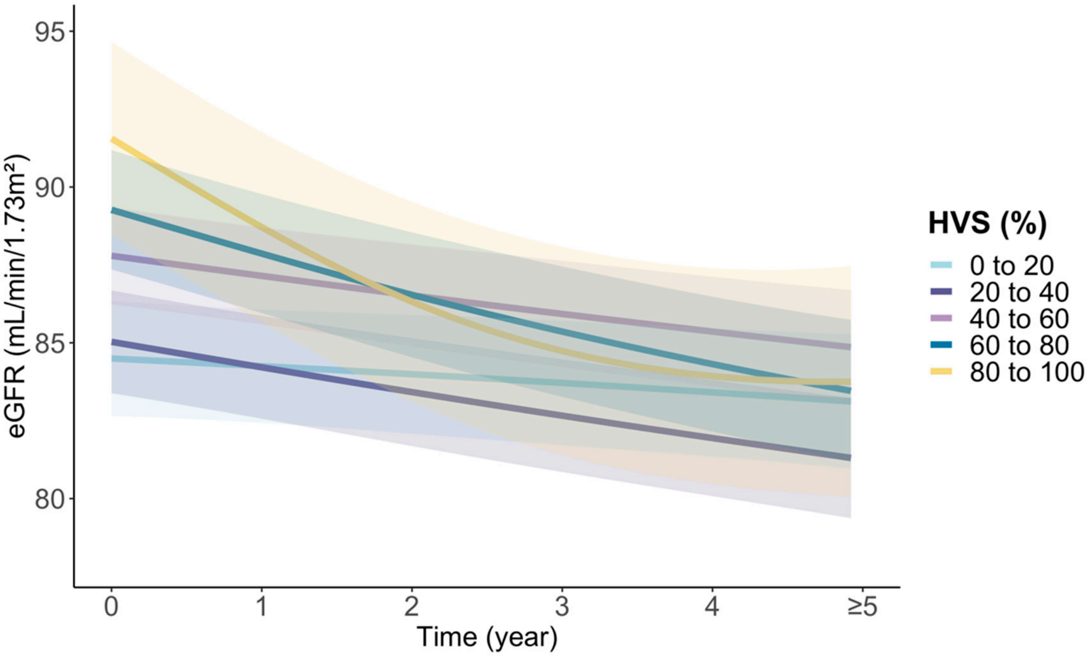

Abbreviations: HVS, glycated haemoglobin A1c score; eGFR, estimated glomerular filtration rate.

Supplementary Figure S2 The covariate balance across HVS categories before and after applying entropy balancing

A

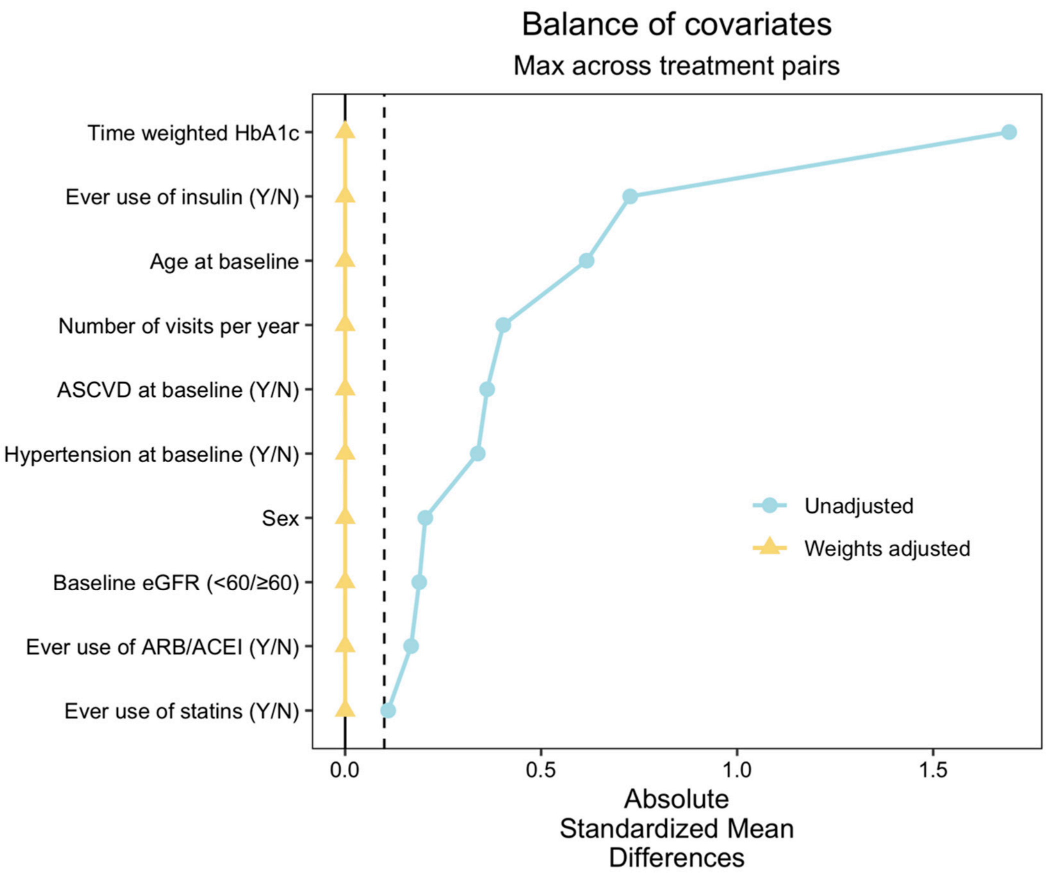

B

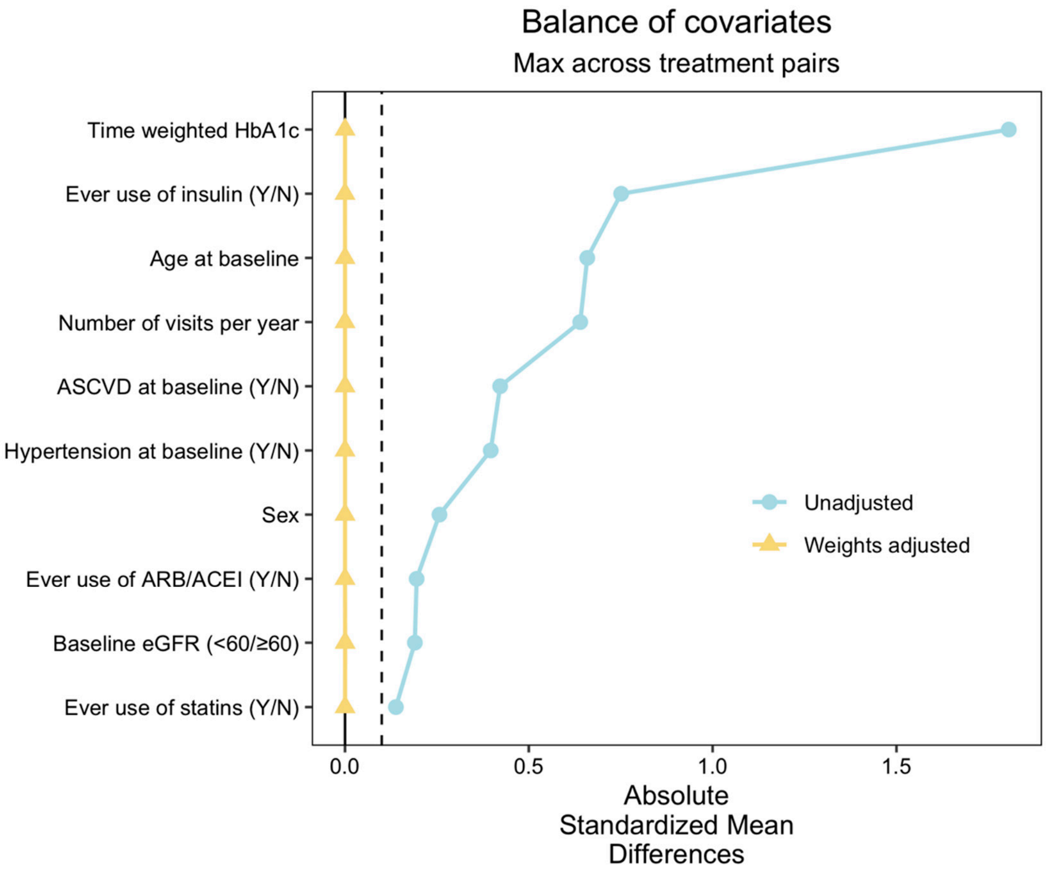

A, assessing the covariate balance for logistic regression of HVS and experiencing a rapid eGFR annual decline.

B, assessing the covariate balance for the linear mixed effects model.

Abbreviations: HVS, glycated haemoglobin A1c variability score; HbA1c, glycated haemoglobin A1c; eGFR, estimated glomerular filtration rate; ASCVD, atherosclerotic cardiovascular disease; ACEI, angiotensin-converting enzyme inhibitor; ARB, angiotensin II, receptor blockers.

Supplementary Figure S3 The flowchart of the selection of study population

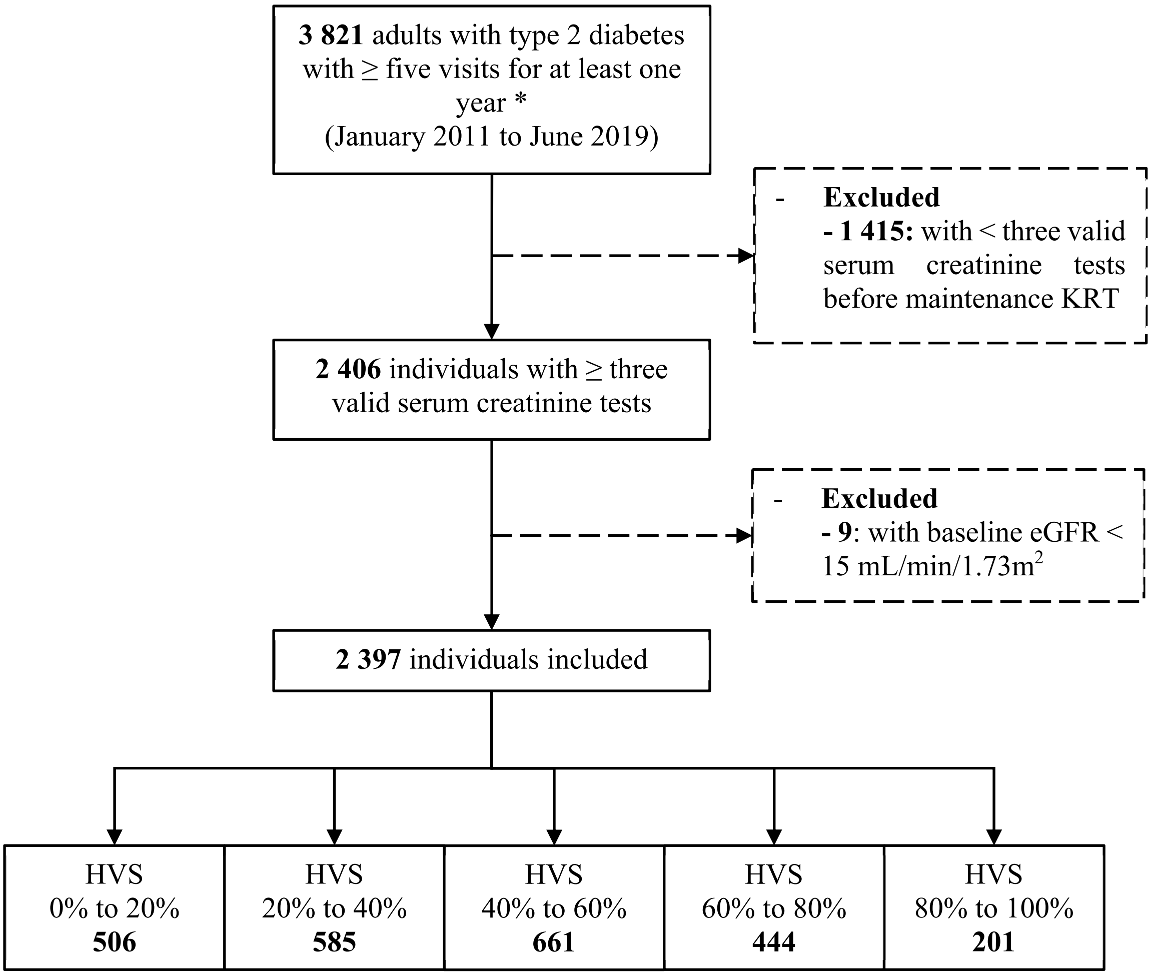

*We recruited adults with type 2 diabetes who had ≥ five outpatient visits for at least one year (from the first visit with HbA1c measure to the last) using electronic medical records of four hospitals from the West China Electronic medical record Collaboration Of DiabEtes (WECODe) outpatient setting from 01 Jan 2011 to 30 Jun 2019.

Abbreviations: HbA1c, glycated haemoglobin A1c; HVS, glycated haemoglobin A1c score; eGFR, estimated glomerular filtration rate; KRT, kidney replacement therapy.

Supplementary Figure S4 Individual baseline eGFR and his/her mean eGFR annual change, stratified by HVS categories

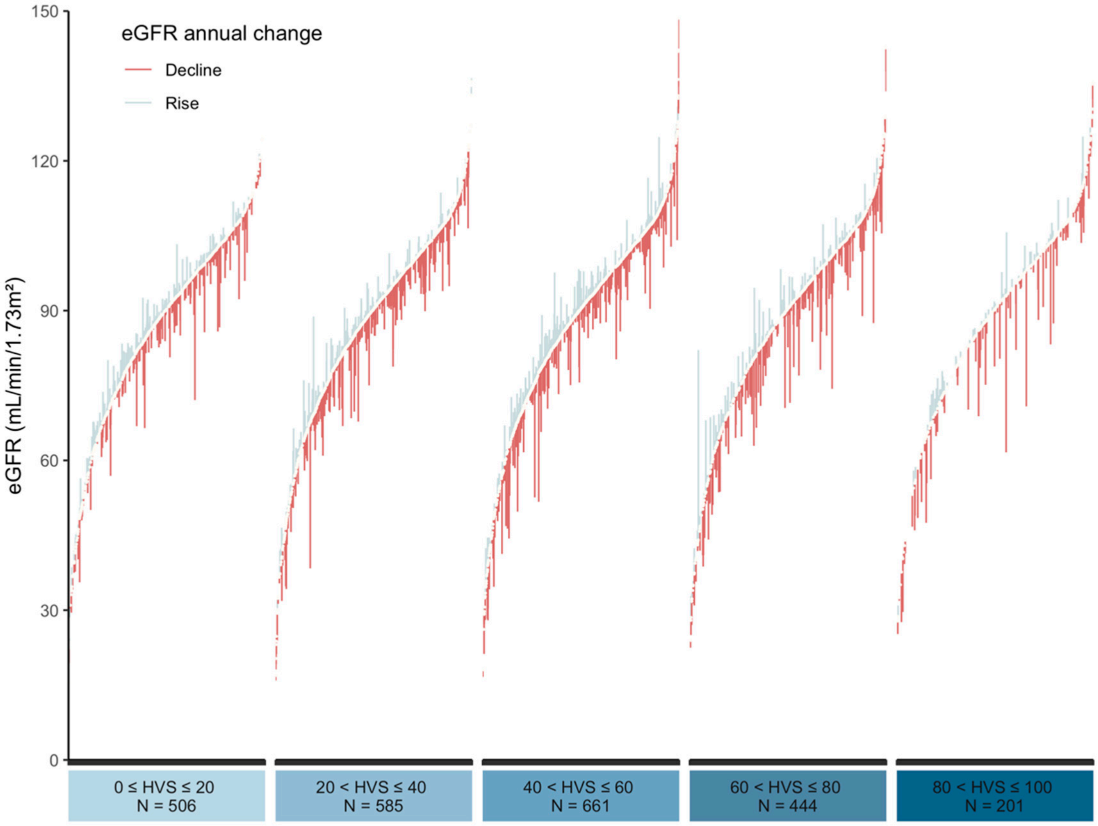

Abbreviations: HVS, glycated haemoglobin A1c variability score; eGFR, estimated glomerular filtration rate.

The unit of HVS is %. The white point represents the baseline eGFR. The length of red line represents the mean annual decline from baseline to end of follow-up. The length of blue one is the mean annual rise from baseline to end of follow-up.

Supplementary Figure S5 Subgroup analyses of the association between HVS and experiencing a rapid eGFR annual decline

Subgroup	HVS category	n / N	Odds ratio (95% CI)
**Sex**			
Female	0 ≤ HVS ≤ 20	10 / 237	
	20 < HVS ≤ 40	19 / 238	1.04 (0.99 to 1.09)
	40 < HVS ≤ 60	17 / 254	1.03 (0.98 to 1.08)
	60 < HVS ≤ 80	29 / 176	1.13 (1.07 to 1.19)
	80 < HVS ≤ 100	11 / 74	1.09 (1.02 to 1.17)
Male	0 ≤ HVS ≤ 20	24 / 269	
	20 < HVS ≤ 40	48 / 347	1.10 (1.04 to 1.16)
	40 < HVS ≤ 60	53 / 407	1.06 (1.00 to 1.12)
	60 < HVS ≤ 80	42 / 268	1.09 (1.03 to 1.16)
	80 < HVS ≤ 100	22 / 127	1.39 (1.29 to 1.50)
**Age**			
Age <60	0 ≤ HVS ≤ 20	21 / 244	
	20 < HVS ≤ 40	33 / 271	1.08 (1.02 to 1.13)
	40 < HVS ≤ 60	36 / 367	1.02 (0.97 to 1.07)
	60 < HVS ≤ 80	42 / 282	1.08 (1.03 to 1.14)
	80 < HVS ≤ 100	16 / 132	1.13 (1.05 to 1.21)
Age ≥60	0 ≤ HVS ≤ 20	13 / 262	
	20 < HVS ≤ 40	34 / 314	1.08 (1.02 to 1.15)
	40 < HVS ≤ 60	34 / 294	1.08 (1.02 to 1.15)
	60 < HVS ≤ 80	29 / 162	1.14 (1.07 to 1.22)
	80 < HVS ≤ 100	17 / 69	1.43 (1.32 to 1.55)
**Ever use of insulin**			
No	0 ≤ HVS ≤ 20	26 / 402	
	20 < HVS ≤ 40	37 / 378	1.03 (0.98 to 1.08)
	40 < HVS ≤ 60	41 / 356	1.04 (0.99 to 1.09)
	60 < HVS ≤ 80	22 / 206	1.04 (0.99 to 1.10)
	80 < HVS ≤ 100	17 / 90	1.27 (1.19 to 1.36)
Yes	0 ≤ HVS ≤ 20	8 / 104	
	20 < HVS ≤ 40	30 / 207	1.15 (1.08 to 1.23)
	40 < HVS ≤ 60	29 / 305	1.06 (0.99 to 1.12)
	60 < HVS ≤ 80	49 / 238	1.21 (1.13 to 1.29)
	80 < HVS ≤ 100	16 / 111	1.24 (1.14 to 1.36)
**Baseline eGFR**			
<60 mL/min/1.73m²	0 ≤ HVS ≤ 20	3 / 61	
	20 < HVS ≤ 40	8 / 78	1.05 (0.93 to 1.19)
	40 < HVS ≤ 60	9 / 73	1.07 (0.95 to 1.20)
	60 < HVS ≤ 80	9 / 46	1.10 (0.96 to 1.25)
	80 < HVS ≤ 100	6 / 15	1.52 (1.28 to 1.79)
≥60 mL/min/1.73m²	0 ≤ HVS ≤ 20	31 / 445	
	20 < HVS ≤ 40	59 / 507	1.08 (1.04 to 1.12)
	40 < HVS ≤ 60	61 / 588	1.04 (1.00 to 1.09)
	60 < HVS ≤ 80	62 / 398	1.11 (1.06 to 1.16)
	80 < HVS ≤ 100	27 / 186	1.23 (1.16 to 1.30)

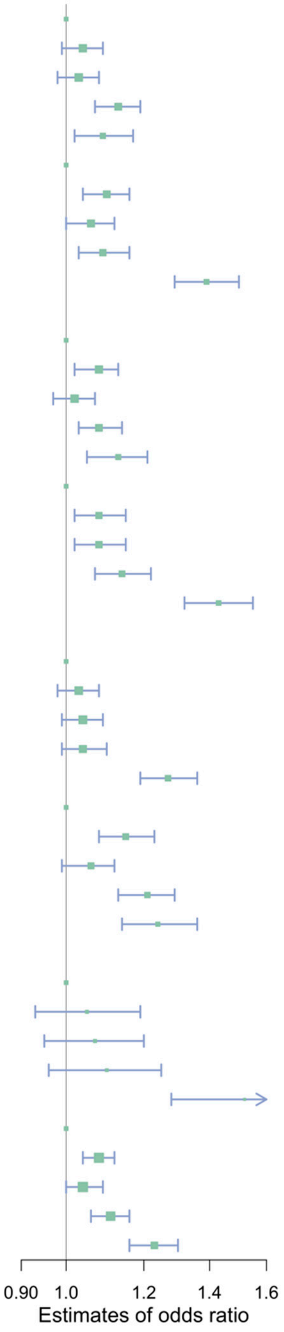

Abbreviations: HVS, glycated haemoglobin A1c score; eGFR, estimated glomerular filtration rate; CI, confidence interval.

A rapid eGFR annual decline is defined as eGFR annual decline at 5 mL/min/1.73m^2/year or more on average from baseline to end of follow-up.

The unit of HVS is %.

Supplementary Figure S6 Subgroup analyses of difference across HVS categories in the mean eGFR slope starting from baseline to end of follow-up

Subgroup	HVS category	N	Slope (95% CI)	Mean difference (95% CI)
**Sex**				
Female	0 ≤ HVS ≤ 20	237	-0.33 (-0.79 to 0.13)	
	20 < HVS ≤ 40	238	-0.68 (-1.07 to -0.30)	-0.35 (-0.96 to 0.25)
	40 < HVS ≤ 60	254	-0.35 (-0.74 to 0.04)	-0.02 (-0.62 to 0.58)
	60 < HVS ≤ 80	176	-1.37 (-1.87 to -0.87)	-1.04 (-1.72 to -0.36)
	80 < HVS ≤ 100	74	-1.87 (-2.71 to -1.02)	-1.54 (-2.50 to -0.57)
Male	0 ≤ HVS ≤ 20	269	-0.34 (-0.80 to 0.13)	
	20 < HVS ≤ 40	347	-0.91 (-1.28 to -0.55)	-0.58 (-1.17 to 0.01)
	40 < HVS ≤ 60	407	-0.75 (-1.09 to -0.40)	-0.41 (-0.99 to 0.17)
	60 < HVS ≤ 80	268	-1.19 (-1.63 to -0.76)	-0.86 (-1.50 to -0.22)
	80 < HVS ≤ 100	127	-2.38 (-3.16 to -1.60)	-2.04 (-2.95 to -1.13)
**Age**				
Age <60	0 ≤ HVS ≤ 20	244	-0.40 (-0.88 to 0.07)	
	20 < HVS ≤ 40	271	-0.75 (-1.15 to -0.36)	-0.35 (-0.97 to 0.27)
	40 < HVS ≤ 60	367	-0.49 (-0.85 to -0.13)	-0.09 (-0.69 to 0.51)
	60 < HVS ≤ 80	282	-1.06 (-1.49 to -0.62)	-0.65 (-1.30 to -0.01)
	80 < HVS ≤ 100	132	-1.62 (-2.41 to -0.83)	-1.21 (-2.14 to -0.29)
Age ≥60	0 ≤ HVS ≤ 20	262	-0.21 (-0.66 to 0.24)	
	20 < HVS ≤ 40	314	-0.86 (-1.21 to -0.50)	-0.64 (-1.22 to -0.07)
	40 < HVS ≤ 60	294	-0.72 (-1.09 to -0.35)	-0.51 (-1.09 to 0.08)
	60 < HVS ≤ 80	162	-1.60 (-2.11 to -1.10)	-1.39 (-2.07 to -0.71)
	80 < HVS ≤ 100	69	-3.02 (-3.86 to -2.18)	-2.81 (-3.76 to -1.85)
**Ever use of insulin**				
No	0 ≤ HVS ≤ 20	402	-0.32 (-0.66 to 0.03)	
	20 < HVS ≤ 40	378	-0.54 (-0.83 to -0.25)	-0.22 (-0.67 to 0.23)
	40 < HVS ≤ 60	356	-0.50 (-0.81 to -0.20)	-0.19 (-0.65 to 0.27)
	60 < HVS ≤ 80	206	-0.64 (-1.05 to -0.23)	-0.32 (-0.86 to 0.21)
	80 < HVS ≤ 100	90	-2.18 (-2.90 to -1.47)	-1.87 (-2.66 to -1.08)
Yes	0 ≤ HVS ≤ 20	104	-0.49 (-1.21 to 0.23)	
	20 < HVS ≤ 40	207	-1.28 (-1.78 to -0.77)	-0.79 (-1.67 to 0.09)
	40 < HVS ≤ 60	305	-0.68 (-1.11 to -0.24)	-0.19 (-1.03 to 0.65)
	60 < HVS ≤ 80	238	-1.89 (-2.41 to -1.36)	-1.40 (-2.29 to -0.51)
	80 < HVS ≤ 100	111	-1.93 (-2.85 to -1.01)	-1.44 (-2.61 to -0.28)
**Baseline eGFR**				
<60 mL/min/1.73m²	0 ≤ HVS ≤ 20	61	-0.59 (-1.67 to 0.50)	
	20 < HVS ≤ 40	78	-0.84 (-1.73 to 0.04)	-0.26 (-1.65 to 1.13)
	40 < HVS ≤ 60	73	-1.23 (-2.15 to -0.31)	-0.64 (-2.06 to 0.77)
	60 < HVS ≤ 80	46	-0.68 (-1.89 to 0.54)	-0.09 (-1.71 to 1.53)
	80 < HVS ≤ 100	15	-3.54 (-5.68 to -1.40)	-2.95 (-5.34 to -0.57)
≥60 mL/min/1.73m²	0 ≤ HVS ≤ 20	445	-0.32 (-0.66 to 0.03)	
	20 < HVS ≤ 40	507	-0.82 (-1.10 to -0.54)	-0.51 (-0.95 to -0.06)
	40 < HVS ≤ 60	588	-0.51 (-0.77 to -0.24)	-0.19 (-0.63 to 0.25)
	60 < HVS ≤ 80	398	-1.31 (-1.65 to -0.97)	-1.00 (-1.48 to -0.51)
	80 < HVS ≤ 100	186	-2.08 (-2.67 to -1.48)	-1.76 (-2.45 to -1.07)

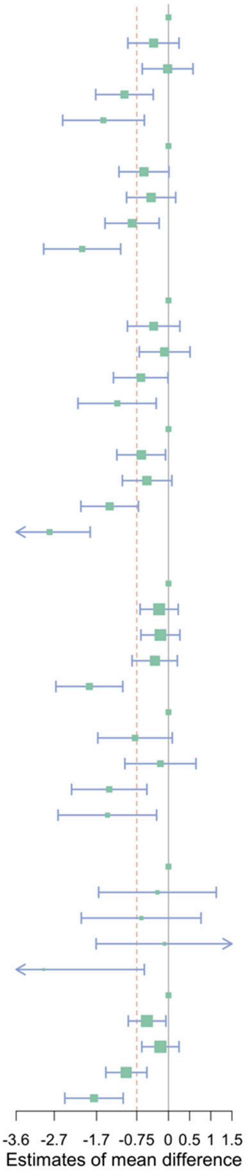

Abbreviations: HVS, glycated haemoglobin A1c score; eGFR, estimated glomerular filtration rate; CI, confidence interval.

The unit of HVS is %.

Supplementary Figure S7 Sensitivity analysis by calculating a mean eGFR slope starting from baseline to either 2-year, 3-year, 4-year, and 5-year follow-up

A

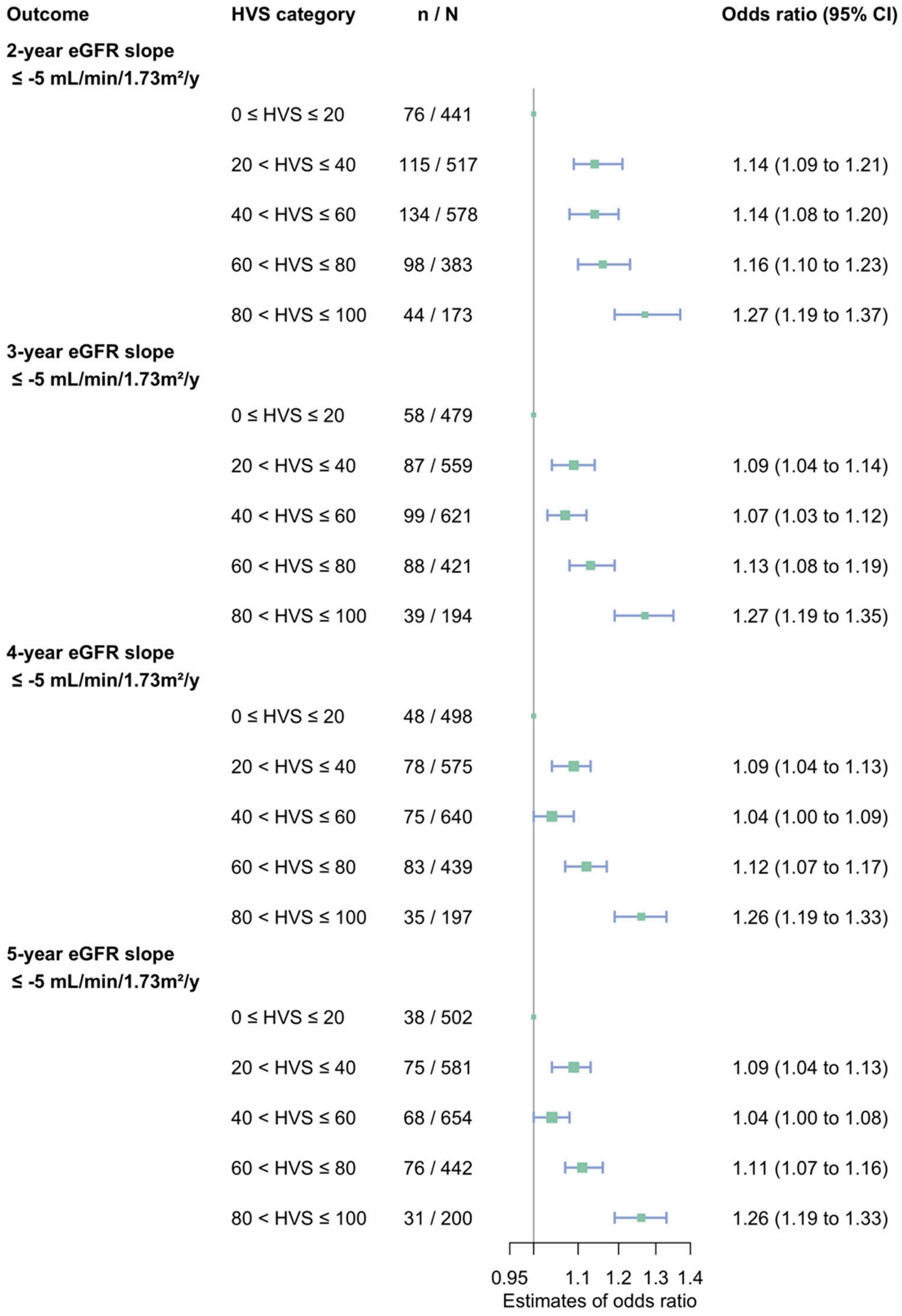

B

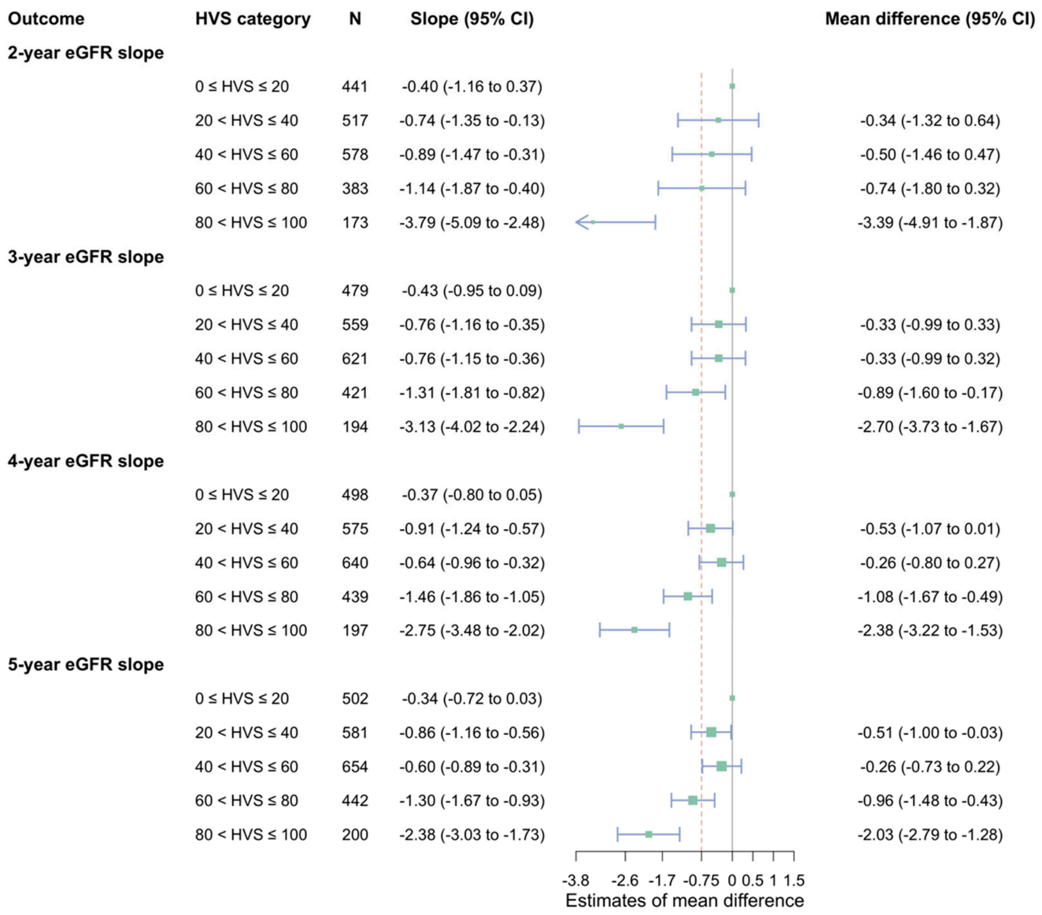

A, odds ratios of HVS categories for experiencing a rapid eGFR annual decline at 5 mL/min/1.73m^2/year or more on average from baseline to either 2-year, 3-year, 4-year, and 5-year follow-up.

B, the difference across HVS categories in the mean eGFR slope from baseline to either 2-year, 3-year, 4-year, and 5-year follow-up.

Abbreviations: HVS, glycated haemoglobin A1c variability score; eGFR, estimated glomerular filtration rate; CI, confidence interval.

The unit of HVS is %.

Supplementary Figure S8 Sensitivity analysis by excluding individuals with the last HbA1c and serum creatinine measured ≥ 90 days apart

A

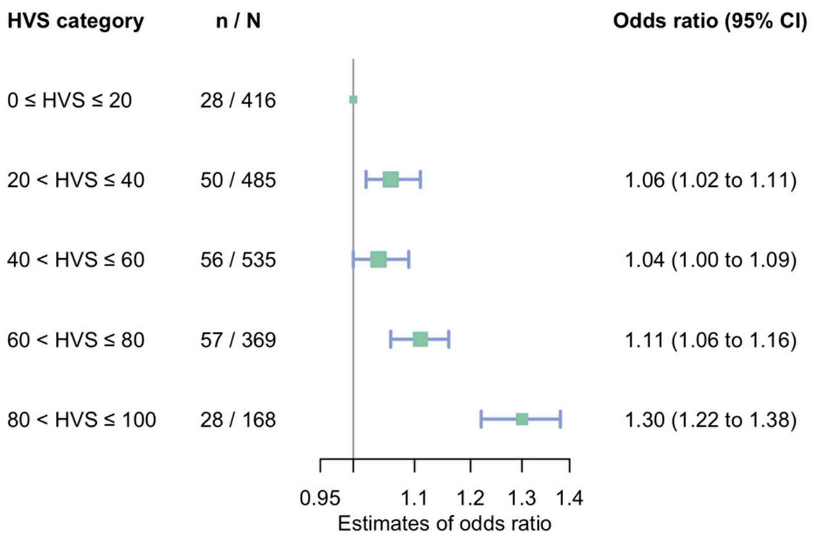

B

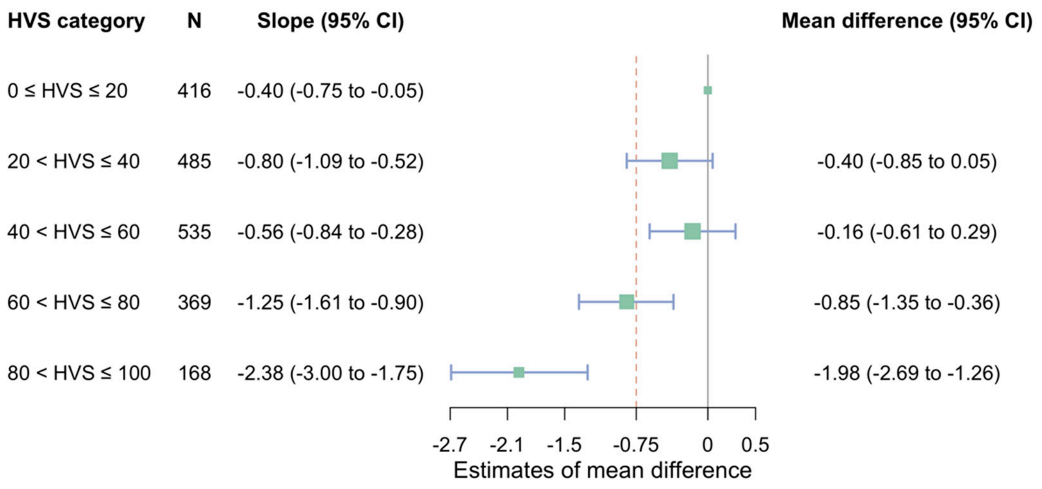

A, odds ratios of HVS categories for experiencing a rapid eGFR annual decline at 5 $mL/min/1.73m^2/year$ or more on average from baseline to end of follow-up.

B, the difference across HVS categories in the mean eGFR slope from baseline to end of follow-up.

Abbreviations: HVS, glycated haemoglobin A1c variability score; CI, confidence interval.

The unit of HVS is %.

Supplementary Figure S9 Sensitivity analysis by adjusting for baseline HbA1c instead of time-weighted average HbA1c when calculating entropy balance weights

A

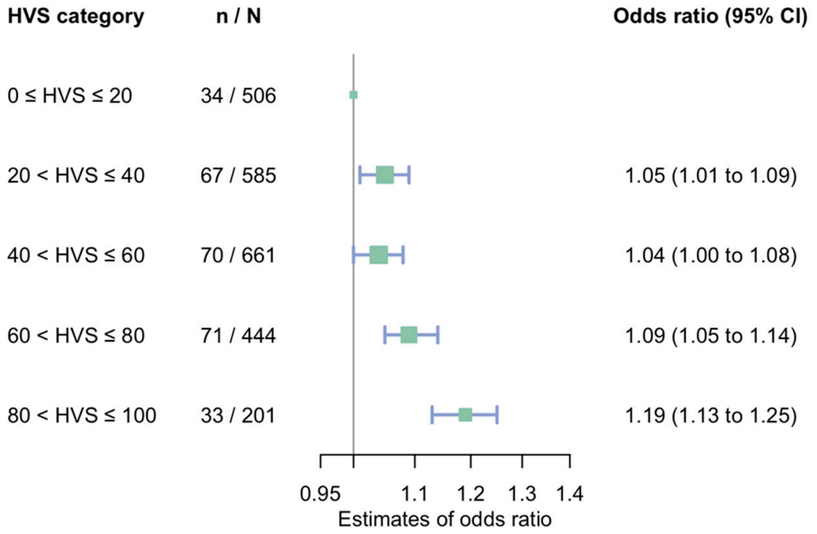

B

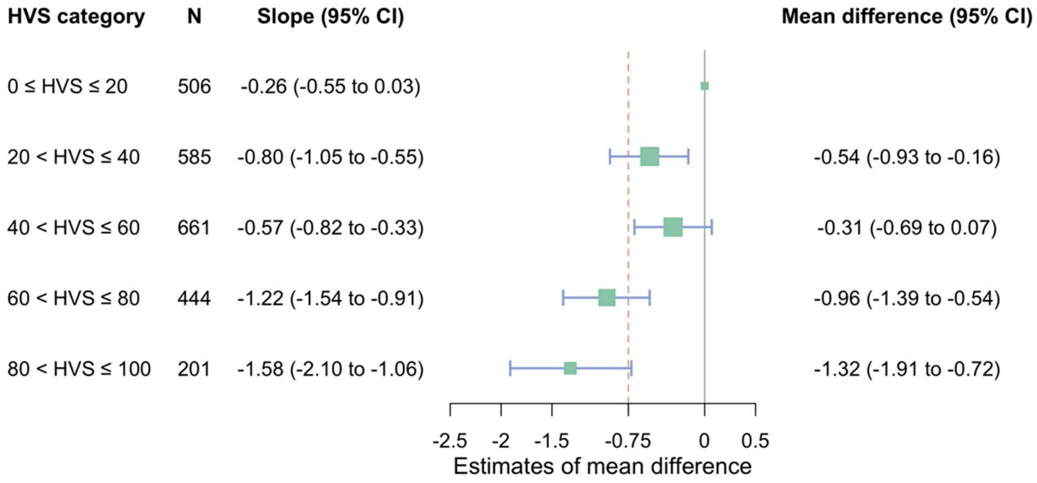

A, odds ratios of HVS categories for experiencing a rapid eGFR annual decline at 5 mL/min/1.73m^2/year or more on average from baseline to end of follow-up.

B, the difference across HVS categories in the mean eGFR slope from baseline to end of follow-up.

Abbreviations: HVS, glycated haemoglobin A1c variability score; CI, confidence interval.

The unit of HVS is %.

Supplementary Figure S10 Sensitivity analysis by excluding individuals with baseline eGFR < 30 mL/min/1.73m^2 instead of eGFR < 15 mL/min/1.73m^2

A

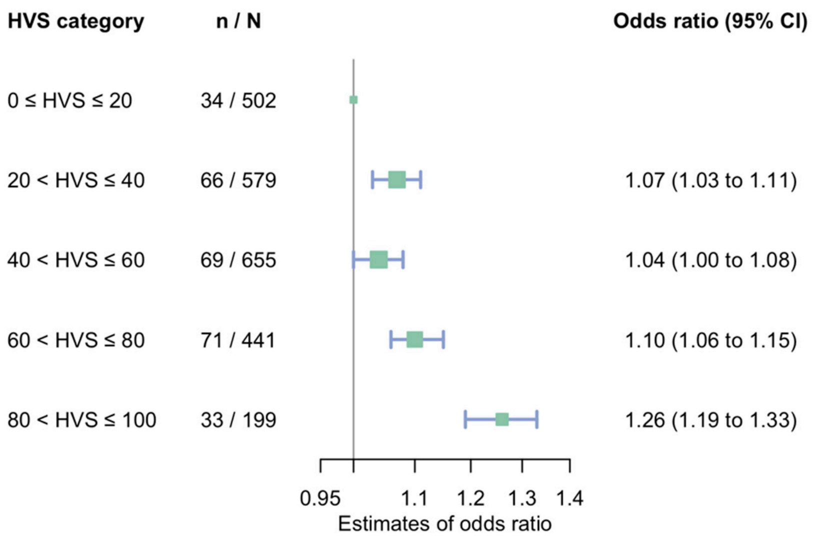

B

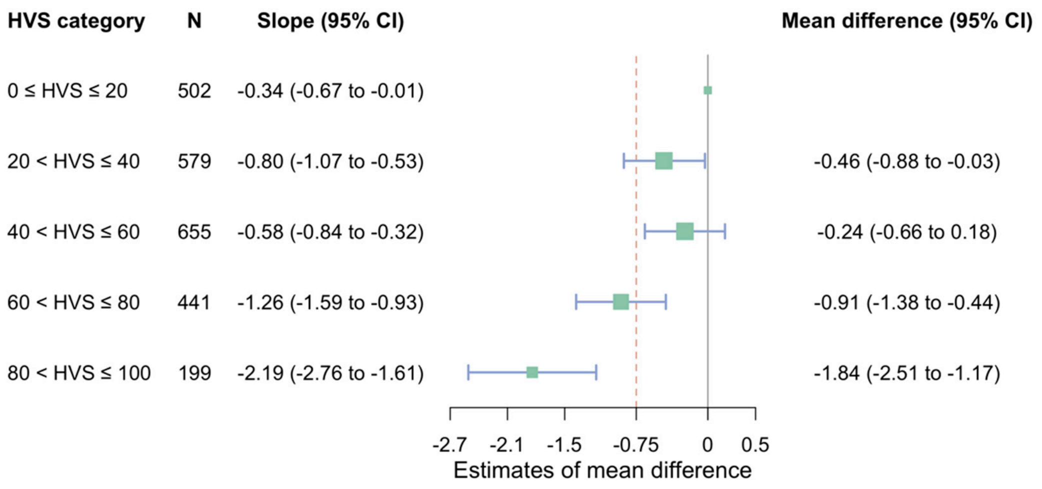

A, odds ratios of HVS categories for experiencing a rapid eGFR annual decline at 5 mL/min/1.73m^2/year or more on average from baseline to end of follow-up.

B, the difference across HVS categories in the mean eGFR slope from baseline to end of follow-up.

Abbreviations: HVS, glycated haemoglobin A1c variability score; CI, confidence interval.

The unit of HVS is %.

Supplementary Figure S11 Sensitivity analysis by excluding individuals receiving any prescription of sodium-glucose cotransporter-2 (SGLT2) inhibitors or glucagon-like peptide-1 (GLP1) receptor agonists during follow up

A

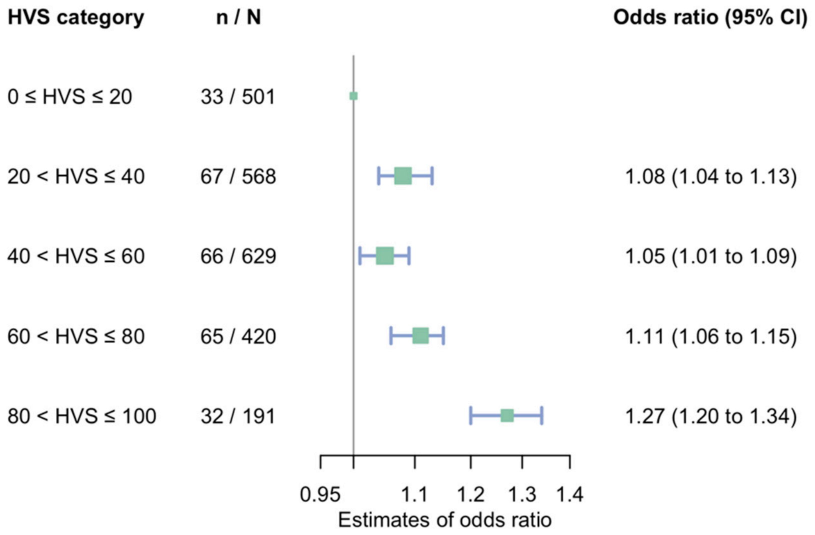

B

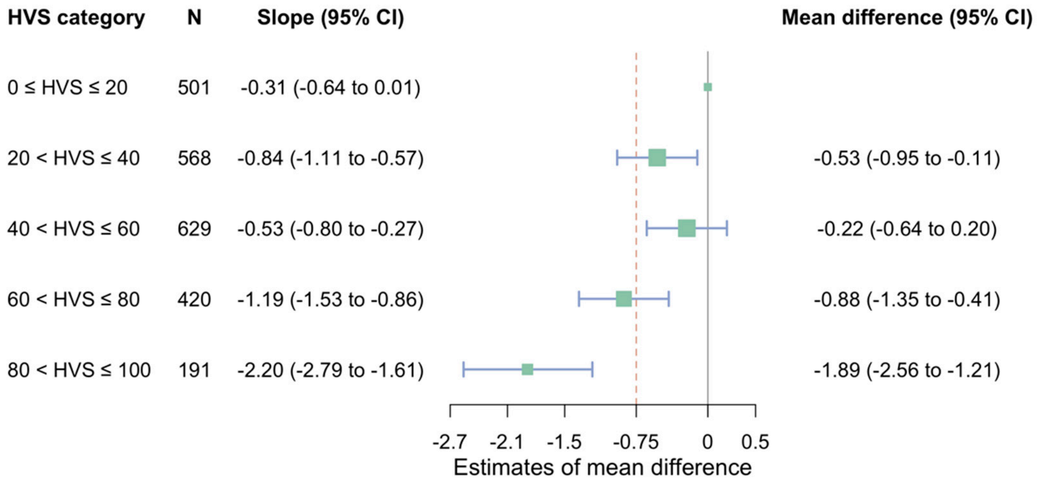

A, odds ratios of HVS categories for experiencing a rapid eGFR annual decline at 5 mL/min/1.73m^2/year or more on average from baseline to end of follow-up.

B, the difference across HVS categories in the mean eGFR slope from baseline to end of follow-up.

Abbreviations: HVS, glycated haemoglobin A1c variability score; CI, confidence interval.

The unit of HVS is %.
